# Solid-phase extraction cartridges with multi-walled carbon nanotubes and effect of the oxygen functionalities on the recovery efficiency of organic micropollutants

**DOI:** 10.1038/s41598-020-79244-8

**Published:** 2020-12-18

**Authors:** Marta O. Barbosa, Rui S. Ribeiro, Ana R. L. Ribeiro, M. Fernando R. Pereira, Adrián M. T. Silva

**Affiliations:** Laboratory of Separation and Reaction Engineering - Laboratory of Catalysis and Materials (LSRE-LCM), Faculdade de Engenharia, Universidade do Porto, Rua Dr. Roberto Frias s/n, 4200-465 Porto, Portugal

**Keywords:** Analytical chemistry, Environmental chemistry, Materials chemistry, Surface chemistry, Environmental sciences

## Abstract

Pristine and functionalized multi-walled carbon nanotubes (MWCNTs) were investigated as adsorbent materials inside solid-phase extraction (SPE) cartridges for extraction and preconcentration of 8 EU-relevant organic micropollutants (with different p*K*a and polarity) before chromatographic analysis of surface water. The recoveries obtained were > 60% for 5/8 target pollutants (acetamiprid, atrazine, carbamazepine, diclofenac, and isoproturon) using a low amount of this reusable adsorbent (50 mg) and an eco-friendly solvent (ethanol) for both conditioning and elution steps. The introduction of oxygenated surface groups in the carbon nanotubes by using a controlled HNO_3_ hydrothermal oxidation method, considerably improved the recoveries obtained for PFOS (perfluorooctanesulfonic acid) and methiocarb, which was ascribed to the hydrogen bond adsorption mechanism, but decreased those observed for the pesticide acetamiprid and for two pharmaceuticals (carbamazepine and diclofenac), suggesting π–π dispersive interactions. Moreover, a good correlation was found between the recovery obtained for methiocarb and the amount of oxygenated surface groups on functionalized MWCNTs, which was mainly attributed to the increase of phenols and carbonyl and quinone groups. Thus, the HNO_3_ hydrothermal oxidation method can be used to finely tune the surface chemistry (and texture) of MWCNTs according to the specific micropollutants to be extracted and quantified in real water samples.

## Introduction

In the last decades, a growing interest has been raised about the fate and effects of a large group of organic micropollutants (OMPs) on the aquatic environment. These pollutants found at trace concentrations (ng L^−1^ to μg L^−1^) can be natural or anthropogenic substances, such as pharmaceutical compounds, pesticides, industrial compounds and steroid hormones^1^. Conventional wastewater treatment plants are not designed to completely remove many of these organic compounds at low concentrations, which are thus discharged into receiving water bodies, including groundwater and surface water (SW), reaching drinking water for human consumption^2^. Other sources of contamination include direct discharge and runoff, namely in the case of industrial compounds, pesticides applied in agriculture, and veterinary pharmaceuticals used for livestock and aquaculture^3,4^. Most of these compounds are pseudo-persistent since their transformation/removal rates are overcome by their continuous release into the environment. Moreover, their recalcitrant character and polarity favours the dispersion and interchange between aquatic compartments^4,5^. The presence of such OMPs in the aquatic environment is considered an important issue in terms of public health safety^6^. Therefore, the monitoring of specific priority substances (PSs, Directive 2013/39) and some contaminants of emerging concern (CECs, Decision 2018/840 and Decision 2020/161) in SW bodies has been recommended within the European Union (EU). The comprehensive identification and quantification of PSs and CECs in freshwater samples is crucial to collect information on their sources, distribution and fate in the environment, to study the effects on ecosystems and human health, and to update the water policy in this field. To achieve this goal, it is important to set up fast, sensitive and reliable analytical methods enabling the determination of a wide range of OMPs typically found at residual levels in aquatic compartments.

Despite the shortcomings of solid phase extraction (SPE), such as the high volumes of organic solvents needed in comparison with miniaturized techniques, time consumption and high cost, this sample preparation technique is still the most employed for preconcentration of OMPs in water matrices due to the efficient removal of interferences, consequent reduction of matrix effects and high enrichment factors and recoveries often yielded^1,7^. SPE is an essential preconcentration step prior to analysis by a sensitive and reproducible analytical technique such as ultra-high performance liquid chromatography-tandem mass spectrometry (UHPLC-MS/MS). The type of sorbent, its structure and interactions with the target analytes play an important role in SPE, reason why many carbon materials have been already reported as good candidates as filling materials for this purpose^8,9^.

Multi-walled carbon nanotubes (MWCNTs) are the most studied carbon materials for environmental applications of SPE. This fact can be attributed to: (i) the unique structure of MWCNTs that enables strong interactions with organic molecules through non-covalent forces (i.e., hydrophobic interactions, hydrogen bonding, π–π stacking, electrostatic forces and van der Waals forces); (ii) their large surface-to-volume ratio; (iii) good thermal and mechanical stability; and (iv) the possibility to control their affinity towards target compounds upon surface functionalization by chemical or physical methods^10^. MWCNTs have been investigated as SPE sorbents for sample preparation of water matrices and to pre-concentrate and extract OMPs such as pesticides (e.g.^11–16^), polycyclic aromatic hydrocarbons^17,18^, industrial compounds^19^, macrolide antibiotics and nonsteroidal anti-inflammatory drugs^20,21^, with recoveries higher than 62%. However, the number of studies dealing with multi-class PSs and CECs with different physicochemical properties are still very limited^20,21^. Moreover, application of functionalized MWCNTs in SPE for extraction of EU-relevant OMPs is even scarcer in the literature. Only three studies were reported, namely for: (i) pentachlorophenol using MWCNTs oxidized with 8.0 mol L^−1^ of HNO_3_^22^; (ii) the industrial compound perfluorooctanesulfonic acid (PFOS) using amino-terminated alkyl-functionalized MWCNTs^19^; and (iii) thirteen pharmaceutical compounds, some of them defined in the EU Decisions (erythromycin, azithromycin and diclofenac), using MWCNTs treated with high concentrations of HNO_3_ (4.0 mol L^−1^), HCl (1.0 mol L^−1^) and KOH (4.0 mol L^−1^)^21^, i.e. having pronounced environmental implications and costs.

In the present work, pristine and modified MWCNTs were investigated as SPE sorbents for the simultaneous extraction of 8 EU multi-class OMPs in SW before UHPLC-MS/MS analysis. The target compounds, namely 5 pesticides (acetamiprid, atrazine, isoproturon, metaflumizone and methiocarb), 2 pharmaceutical compounds (carbamazepine and diclofenac), and one industrial compound (PFOS) were strategically selected due to their high frequency of detection and/or their high levels of concentration in water matrices observed during the monitoring sampling campaigns performed by our research group in the last years^2,6^ and, in the specific case of metaflumizone due to its presence in the recently 3rd Watch List (Decision 2020/1161). A set of experiments was performed using pristine MWCNTs to study the parameters that influenced the extraction efficiency of the 8 OMPs spiked in SW samples, namely the sample pH and volume, the elution and extraction solvent and respective volumes, and the amount of MWCNTs packed in the cartridge. After optimizing these parameters, the cartridge packed with MWCNTs and the commercial cartridge Oasis HLB were compared, in terms of extraction efficiency, reusability, and costs. Then, we attempted to investigate a HNO_3_ hydrothermal oxidation methodology reported by our group^23^ to obtain a series of MWCNTs with meticulously introduced surface oxygen functionalities. This methodology allows the fine control of the type and amount of surface groups introduced on carbon materials by adjusting the concentration of oxidizing agent employed in the treatment (HNO_3_ concentration in the range 0.01–0.30 mol L^−1^), as determined by different characterization techniques. This distinctive feature allowed establishing correlations between both the synthesis conditions and the oxygen-containing surface functionalities introduced on the MWCNTs; and the type and amount of those functionalities and the recoveries obtained for the 8 target OMPs; while employing much lower concentrations of oxidizing agent than those previously reported with similar hydrothermal methodologies^23–26^. Therefore, the novelty of this study relies on (i) the development of a systematic study, upon application of a controlled HNO_3_ hydrothermal oxidation methodology to pristine MWCNTs; but also on (ii) the use of ethanol as elution solvent in the SPE procedure, when using MWCNT cartridges; and (iii) the study of metaflumizone for the first time in real water compartments.

## Experimental section

### Chemicals and materials

MWCNTs (NC3100, powder) with an average diameter of 9.5 nm, average length of 1.5 µm and > 95% purity were obtained from Nanocyl SA (Sambreville, Belgium). All reference standards (acetamiprid, atrazine, carbamazepine, diclofenac sodium, isoproturon, metaflumizone, methiocarb and PFOS; > 98% purity) and deuterated compounds used as internal standards (acetamiprid-d3, atrazine-d5, diclofenac-d4, fluoxetine-d5 and methiocarb-d3) were purchased from Sigma-Aldrich (Steinhein, Germany). The physicochemical properties of the target compounds can be found in Table S1. Methanol and acetonitrile (MS grade), ethanol (HPLC grade), and hydrochloric acid were obtained from VWR International (Fontenay-sous-Bois, France). Individual stock solutions of 1000 mg L^−1^ of each reference and internal standard were prepared in methanol, ethanol or acetonitrile, depending on their solubility. Two ethanolic working solutions containing the 8 target compounds (2.5 mg L^−1^) and the 5 internal standards (5.0 mg L^−1^) were prepared by dilution of the individual stocks. Sulfuric acid and sodium hydroxide were obtained from Merck (Darmstadt, Germany). Sodium chloride was purchased from José Manuel Gomes dos Santos. Ultrapure water was supplied by a Milli-Q water system. Oasis HLB (Hydrophilic–Lipophilic-Balanced) cartridges (150 mg, 6 mL) were obtained from Waters (Milford, MA, USA), and the empty SPE cartridges (6 mL) with two frits (20 µm) (Bond Elut) were purchased from VWR International (Fontenay-sous-Bois, France). pH measurements were performed with a pHenomenal pH 1100L pH meter (VWR, Germany).

### Surface functionalization of MWCNTs

Hydrothermal oxidation of the pristine MWCNTs was performed in a Teflon-lined stainless-steel autoclave (Mod. 4748, Parr Instruments, USA) with 125 mL of capacity, following the experimental procedure described elsewhere^27^. 75 mL of a HNO_3_ solution (concentration in the range 0.01–0.30 mol L^−1^) was transferred to a PTFE vessel and 0.2 g of the pristine MWCNTs was loaded. The PTFE vessel was placed into the stainless-steel autoclave, which was sealed and placed in an oven at 200 °C for 2 h. After this time, the autoclave was allowed to cool down until ambient temperature. The recovered material was washed several times with distilled water until a neutral pH of the rinsing water was attained, and then dried overnight at 120 °C. Additionally, a blank hydrothermal treatment with distilled water instead of the HNO_3_ solution was performed. The resulting materials were labelled as MWCNT followed by a subscript number corresponding to the concentration of HNO_3_ employed in the hydrothermal treatment in mol L^−1^ (i.e., MWCNT_0_, MWCNT_0.01_, MWCNT_0.05_, MWCNT_0.1_, MWCNT_0.2_, and MWCNT_0.3_).

### Characterization of MWCNTs

Temperature programmed desorption (TPD) was performed in a fully automated AMI-300 Catalyst Characterization Instrument (Altamira Instruments), equipped with a quadrupole mass spectrometer (Dymaxion, Ametek), as described elsewhere^27^. Briefly, TPD is a well-established advanced characterization technique assuming that all oxygen-containing surface groups are decomposed into CO_2_ and CO upon heating under controlled operating conditions^28^. In this case, a low heating rate of 10° C min^−1^, and a high helium flow of 25 cm^3^ min^−1^ were set to minimize secondary reactions during the experiments^26,28^. The mass signals m/z = 28 and 44 were monitored during the thermal analysis, the corresponding TPD spectra being obtained. CO and CO_2_ were calibrated at the end of each analysis with the respective gases. The concentrations of the different oxygen containing surface groups were then obtained by deconvolution analysis of the CO_2_ and CO TPD spectra using a procedure established by our group^28,29^. Accordingly, the peaks in the CO_2_ TPD spectra were assigned to diverse functional groups, namely strongly acidic carboxylic acids (SA), less acidic carboxylic acids (LA), carboxylic anhydrides (CAn), and lactones (Lac). Similarly, the peaks in the CO TPD spectra were assigned to carboxylic anhydrides (Can), phenols (Ph), carbonyls and quinones (CQ), and basic surface groups (Bas), such as pyrones and chromenes. The width at half-height (W) of the peak was taken the same for Can and Lac in the CO_2_ spectra, and the same W was considered for Ph and CQ in the CO spectra whenever peak shoulders were unclear. Thermogravimetric analysis (TGA) was performed in a Netzsch STA 490 PC/4/H Luxx thermal analyser, in which the powder sample was heated from 50 to 900 °C at 10 °C min^−1^, under an inert (N_2_) gas flow. Regarding TPD and TGA analysis, selected experiments were performed in duplicate, the standard deviations (SD) never exceeding the values given in the caption of Fig. 3. Textural properties were determined from N_2_ adsorption–desorption isotherms at − 196 °C, as described in our previous work^23^, and included specific surface area (*S*_BET_), non-microporous specific surface area (*S*_meso_), micropore volume (*V*_micro_) and total pore volume (*V*_total_). The pH at point of zero charge (pH_PZC_) was obtained by pH drift tests^27^.

### MWCNTs SPE procedure

Commercial cartridges Oasis HLB were used for comparison purposes in this study (Text S1). After preparing the cartridges with 50 mg of each adsorbent (Figure S1), the SPE protocol previously optimized (Text S2) for pristine MWCNTs (NC3100) was performed. Briefly, ethanol (4 mL) and ultrapure water (4 mL) were used to condition and equilibrate the cartridge at a flow rate of 1 mL min^−1^. 500 mL of blank or spiked (200 ng L^−1^ of each target compound) SW sample previously acidified to pH 3 was loaded at 10 mL min^−1^. 4 mL of ultrapure water was then added in the washing step, followed by 45 min of vacuum drying. For the elution step, 4 mL of ethanol was used and, after evaporation, the filtered reconstituted ethanolic extracts were analysed by UHPLC-MS/MS. All experiments were performed in triplicate and relative standard deviation (RSD) were estimated. For details on the SPE procedure, please see Supplementary Material (Text S1 and S2; Figure S1).

### Evaluation of the SPE recovery efficiency

The recovery efficiency (%) is the most important parameter supporting the selection of the optimal conditions for a given SPE procedure. Therefore, the performance of the off-line SPE method was assessed considering the recovery efficiency for the 8 target analytes under study. The recovery was calculated as the ratio of the peak areas obtained for extracted spiked sample (A) and the peak areas of the post-spiked extracted sample (B), as described in Figure S2 and Eq. (1).

1 $$Recovery \;efficiency \left(\%\right)=100 \times \left(A/B\right)$$

Since the matrix effect is considered the same in both A and B, and thus not accounted for, this approach allows evaluating exclusively the recovery promoted by the adsorbent material. Total Ion Current (TIC) chromatograms of the 8 target OMPs (200 ng L^−1^) after SPE of a spiked sample and after post-spiking a blank extract using original MWCNT packed cartridges are showed in Figure S3 a and b.

### UHPLC–MS/MS method

A Shimadzu Corporation UHPLC-MS/MS (Tokyo, Japan) consisting of a Nexera UHPLC (two chromatographic pumps LC-30AD with a degasser DGU-20A 5R, an autosampler SIL-30AC, an oven CTO-20AC, and a system controller CBM-20A with a Shimadzu LC Solution Version 5.41SP1 software), and a Ultra Fast Mass Spectrometry series LCMS-8040 triple quadrupole mass spectrometer, was used for SW analysis. The chromatographic separation of the target compounds was performed by using a column Kinetex XB-C18 100 Å (100 × 2.1 mm i.d.; particle diameter of 1.7 μm) acquired to Phenomenex, Inc. (Torrance, CA, USA) operating under gradient mode of flow of the mobile phase water/ethanol (50/50, v/v). The column oven temperature was set at 35 °C. The autosampler temperature was set at 15 °C and the injection volume was 5 μL. The MS settings were: 2.5 dm^3^ min^−1^ of nebulizing gas flow, 12.5 dm^3^ min^−1^ of drying gas flow, capillary voltage of 0.5 kV, 400 °C and 250 °C for source and desolvation temperatures, argon at 230 kPa as CID gas. The quantification and confirmation of the identity of each analyte was performed by selected reaction monitoring (SRM). Along with the retention time of the analyte, the transition between the precursor ion and the most abundant fragment ion (SRM1) was used for quantification and the ratio between SRM1 and the transition between the precursor ion and the second most abundant fragment ion SRM2 was used for identity confirmation. All the analytical parameters used, namely SRM instrument parameters, retention time, linearity, and limits of detection and quantification, are detailed in the Supplementary Material (Tables S2 and S3).

### Sample collection

SW samples (pH = 6.5 ± 0.1) were collected from Cavalum River (tributary of the Sousa River) located in Penafiel (40 km from Porto, Portugal). Samples were stored in amber glass bottles (1 L) at 4 °C until extraction, which was performed within 24 h. Before SPE, all samples were filtered through 1.2-μm glass-fiber filters (47 mm GF/C, Whatmam, Maidstone, United Kingdom) and the pH was adjusted using sulfuric acid or sodium hydroxide solutions, according to the SPE procedure (“MWCNTs SPE procedure” section, Texts S1 and S2).

## Results and discussion

### *Optimization of SPE procedure with *pristine MWCNTs (NC3100) *cartridges*

In order to study the performance of pristine MWCNTs (NC3100) as SPE adsorbent for the simultaneous enrichment of the 8 target EU OMPs with different p*K*a and polarity range, the main experimental conditions affecting the extraction efficiency were optimized, namely the sample pH and volume, the elution and extraction solvent and respective volumes, and the amount of MWCNTs packed in the cartridge.

**Figure 1:**
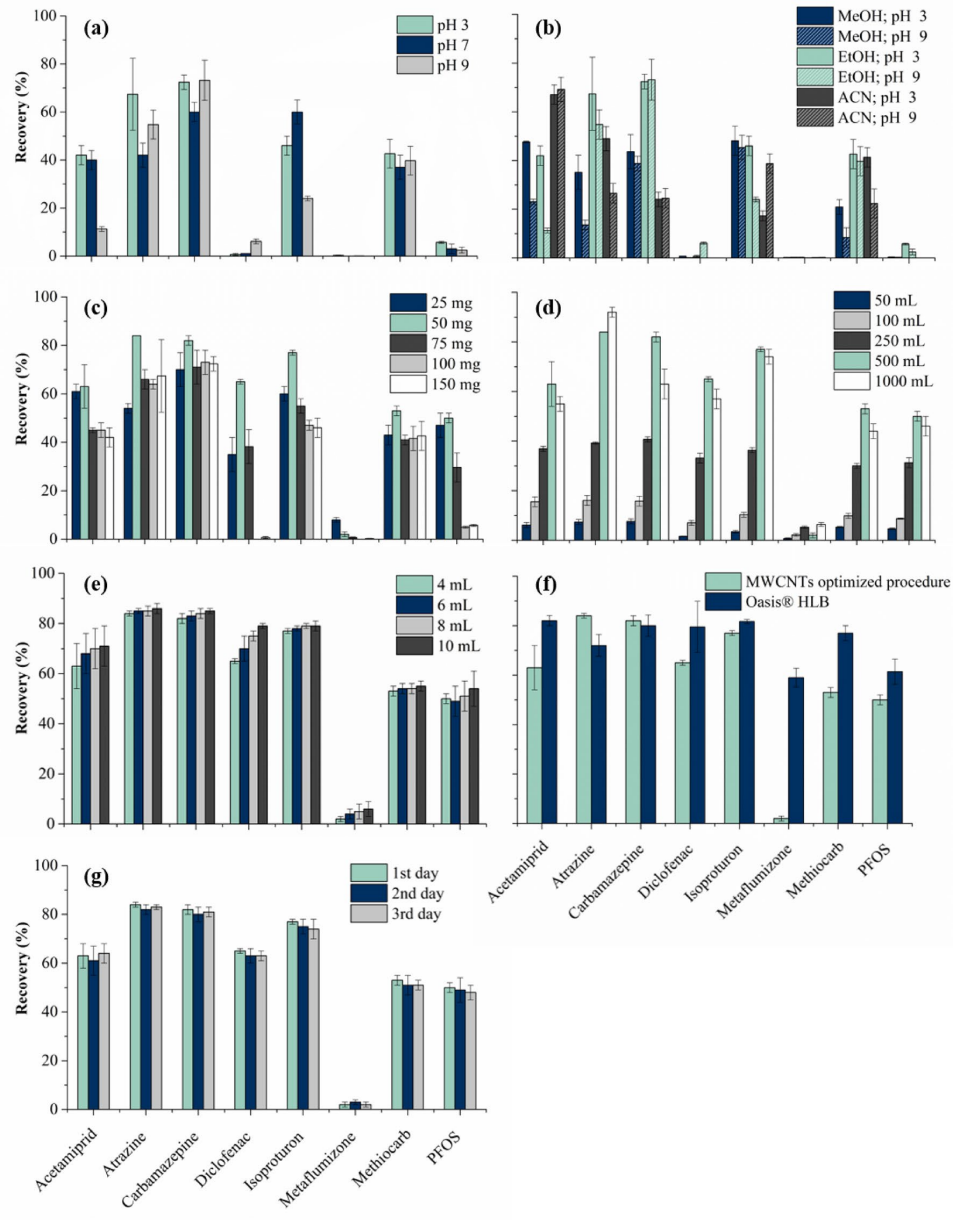
Recoveries obtained for micropollutants (200 ng L^−1^ each), using: (**a**) different pH (3, 7 and 9) (fixed conditions: cartridges packed with 150 mg of MWCNTs, 500 mL of SW and 4 mL of ethanol as a solvent); (**b**) different solvents (4 mL of methanol, ethanol or acetonitrile) and pH (3 and 9) (fixed conditions: cartridges packed with 150 mg of MWCNTs, 500 mL of SW and 4 mL of solvent); (**c**) cartridges packed with different amounts of MWCNTs (25–150 mg) (fixed conditions: pH 3, 500 mL of SW and 4 mL of ethanol as a solvent); (**d**) different volumes (50–1000 mL) of SW (fixed conditions: cartridges packed with 50 mg of MWCNTs, pH 3 and 4 mL of ethanol as a solvent); (**e**) using different volumes (4–10 mL) of ethanol as elution solvent (fixed conditions: cartridges packed with 50 mg of MWCNTs, 500 mL of SW, pH 3); (**f**) MWCNT optimized cartridge (50 mg) and commercial cartridge Oasis HLB (experiments performed with 500 mL of SW samples (pH 3) and using ethanol as solvent (4 mL); and (**g**) recoveries obtained for micropollutants (200 ng L^–1^ each), extracting 500 mL of SW (pH 3) with 4 mL of ethanol as solvent, during consecutive reuse cycles performed with the same cartridge packed with MWCNTs (50 mg); n = 3 (RSD is represented as error bars).

Regarding the sample pH (3, 7 or 9), it determines the state of the target micropollutants in solution as ionic or molecular form, directly affecting the recovery efficiency of the process. When using ethanol as solvent (Fig. 1a), an acidic pH enabled higher recoveries of acetamiprid, atrazine, methiocarb and the industrial compound PFOS, while the recovery of the pharmaceutical compounds carbamazepine and diclofenac performed better at alkaline pH. Neutral pH led to lower recoveries, except for isoproturon. Methanol and acetonitrile were also tested as solvents (Fig. 1b), but ethanol (as conditioning and elution solvent) and an acidic sample pH allowed similar or slightly higher recoveries for most of the target compounds, in comparison with the other studied conditions. Moreover, ethanol is considered an eco-friendly (and greener) solvent^30^, and thus selected for the next experiments. Different amounts of the adsorbent material packed in the SPE cartridge (between 25 and 150 mg) were then investigated (Fig. 1c), and the highest recoveries for the target compounds were obtained when using cartridges packed with 50 mg of MWCNTs (except in the case of the pesticide metaflumizone). Lower recoveries were obtained when using amounts below 50 mg of MWCNTs, which may be due to the limited adsorption capacity of this carbon material at these conditions. Lower recoveries were also obtained for amounts above 50 mg of MWCNTs, which may be explained by a lower desorption of OMPs from MWCNTs during the elution step. Bearing this in mind, 50 mg was considered the optimum amount of MWCNTs, and thus selected for the following experiments.

In what concerns the volume of the SW sample (Fig. 1d), the higher extraction efficiencies were obtained for the majority of the compounds when using a sample volume of 500 mL (except for atrazine and metaflumizone). The sample volume is expected to be directly proportional to the sample preparation enrichment factor (i.e., the ratio between the sample volume and the volume of reconstitution). However, the recoveries obtained for most compounds decreased when the sample volume increased from 500 to 1000 mL. This phenomenon may be ascribed to the SPE breakthrough volume, which is the highest sample volume that allows the maximum extraction efficiency, as observed in previous works^3,6^. Using the optimum volume of SW sample for most OMPs (500 mL), different volumes of eluent (4–10 mL) were then tested (Fig. 1e). The recoveries obtained for the 8 OMPs under study slightly increased with the volume of ethanol used in the elution step, a lower volume of eluent being selected for the next experiments (i.e. lower costs) since the recoveries obtained were quite similar.

The reusability of the MWCNT cartridge is confirmed in Fig. 1g, similar recoveries being obtained in three consecutive cycles. Moreover, the recoveries achieved (> 60%) for 5 of these EU multi-class OMPs analyzed simultaneously (acetamiprid, atrazine, carbamazepine, diclofenac and isoproturon), using a low amount of adsorbent (50 mg of MWCNTs for 500 mL of SW samples at pH 3) and a conditioning and elution solvent considered “green” (ethanol—4 mL), were comparable to those reported in the literature using more toxic solvents and a single compound or specific class of compounds (Table S5). Thus, the next step was to functionalize the MWCNTs in order to investigate the influence of the surface chemistry on the performance of this analytical tool.

#### Comparison of optimized SPE procedures for MWCNT and commercial cartridges

The comparison of enrichment performance of the MWCNT cartridge previously optimized and the commercial cartridge Oasis HLB was performed with SW samples. The optimized SPE methodology was applied and the recoveries of the 8 target micropollutants (spiked at 200 ng L^−1^ each) were obtained (Fig. 1f). A recovery higher than 60% was achieved for 3 pesticides (acetamiprid, atrazine and isoproturon) and the 2 pharmaceutical compounds (carbamazepine and diclofenac) when using both MWCNTs and commercial cartridges. However, the commercial cartridge Oasis HLB gave recovery values also higher than 60% for the industrial compound (PFOS) and the other 2 pesticides (metaflumizone and methiocarb). Except for metaflumizone, the overall recovery of the other 7 micropollutants was similar.

The textural properties of MWCNTs and Oasis HLB were investigated through N_2_ adsorption–desorption isotherms, as described in “Characterization of MWCNTs” section. The results revealed that these materials have different porous characteristics (Table S4). Although the total pore volume (*V*_total_) is similar for both adsorbents (1.264 and 1.284 cm^3^ g^−1^ for MWCNTs and Oasis HLB, respectively), the specific surface area (*S*_BET_) of Oasis HLB (756 m^2^ g^−1^) is ca. 3.8-fold higher than that of MWCNTs (198 m^2^ g^−1^). Consequently, the average pore diameter (*d*_pore_) of MWCNTs (25.5 nm) is almost fourfold higher than that of Oasis HLB (6.8 nm). Interestingly, the sorbent load in each cartridge Oasis HLB is 3 times higher than that of the cartridges packed with MWCNTs, which have a *S*_BET_ 3.8-fold lower. However, the SPE performances obtained for MWCNTs (Fig. 1f) cannot be explained by one unique parameter. Instead, the adsorption followed by elution/desorption of the organic micropollutants result from the interplay of many factors: the textural properties of the adsorbent material (Table S4); the functional groups of the adsorbent and of the organic pollutants; the hydrophobic interactions between the target micropollutants (log *K*_OW_ of each target compound can be found in Table S1) and MWCNTs, and other adsorption mechanisms; the morphology of the adsorbent material; and the sample characteristics (for example, the dissolved organic matter present in the water matrix)^10^.

In addition to the analytical performance of the SPE procedure, it is important to take into consideration the cost of the adsorbent material. Considering 2020 retail prices, each commercial cartridge Oasis HLB costs around 8 euros, while the whole cost associated to each cartridge packed with 50 mg of MWCNTs amounts to ca. 2 euros (including the empty polypropylene cartridge and two frits). This represents a possible cost reduction of 75%. Furthermore, these MWCNT cartridges are reusable, while commercial cartridges often are single-use disposable devices^6,20^.

### Textural and surface chemistry characterization of MWCNTs

**Figure 2:**
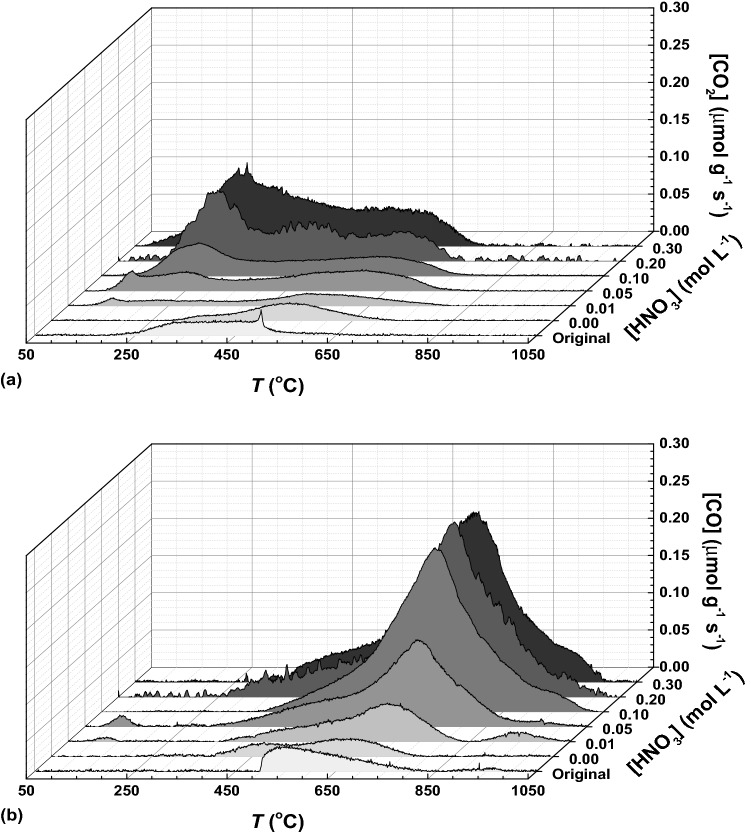
TPD spectra of MWCNTs subjected to hydrothermal treatment with different HNO_3_ concentrations: **(a)** CO_2_ and **(b)** CO evolution with temperature.

The type and overall amount of oxygen-containing surface groups were determined by TPD analysis, as described in “Characterization of MWCNTs” section. The CO_2_ and CO TPD spectra of the hydrothermally treated MWCNTs (HNO_3_ concentration in the range 0.01–0.30 mol L^−1^) are shown in Fig. 2a,b, respectively. For comparison, the TPD profiles determined for the pristine MWCNTs (original) and for MWCNTs after hydrothermal treatment with water (i.e., [HNO_3_] = 0) are also included. The total amount of surface groups (released as CO_2_ and CO) and the corresponding oxygen content (calculated from the total amounts of CO_2_ and CO) are summarized in Table 1.

**Table 1:** Properties of MWCNTs subjected to hydrothermal treatment with different HNO_3_ concentrations: amounts of CO_2_ and CO released by TPD, [CO/CO_2_] ratio, percentage of oxygen obtained from the analysis of the TPD spectra (assuming that all the surface oxygen is released as CO and/or CO_2_), amount of volatiles (determined by TGA), amount of carboxylic acids (CA; corresponding to the sum of SA and LA, as determined by TPD), pH at the point of zero charge (pH_PZC_), specific surface area (*S*_BET_), non-microporous specific surface area (*S*_meso_) and total pore volume (*V*_total_).

[HNO_3_] (mol L^−1^)	Parameters									
	[CO_2_] (μmol g^−1^)	[CO] (μmol g^−1^)	O (wt%)	[CO]/[CO_2_]	Volatiles (wt%)	[CA] (μmol g^−1^)	pH_PZC_	S_BET_ (m^2^ g^−1^)	S_meso_ (m^2^ g^−1^)	V_total_ (cm^3^ g^−1^)
Original MWCNT	59	72	0.3	1.2	2.87	n.d.	6.9	198	198	1.264
0 (Blank)	59	72	0.3	1.2	2.92	5	6.9	188	188	1.581
0.01	71	150	0.5	2.1	3.41	20	6.7	202	202	1.148
0.05	156	327	1.0	2.1	3.75	54	6.1	229	204	1.115
0.10	178	590	1.5	3.3	4.41	93	5.5	250	237	1.566
0.20	304	656	2.0	2.2	4.80	152	5.2	261	255	1.669
0.30	392	671	2.3	1.7	5.12	225	5.0	262	262	2.193

n.d.: Not determined.

**Figure 3:**
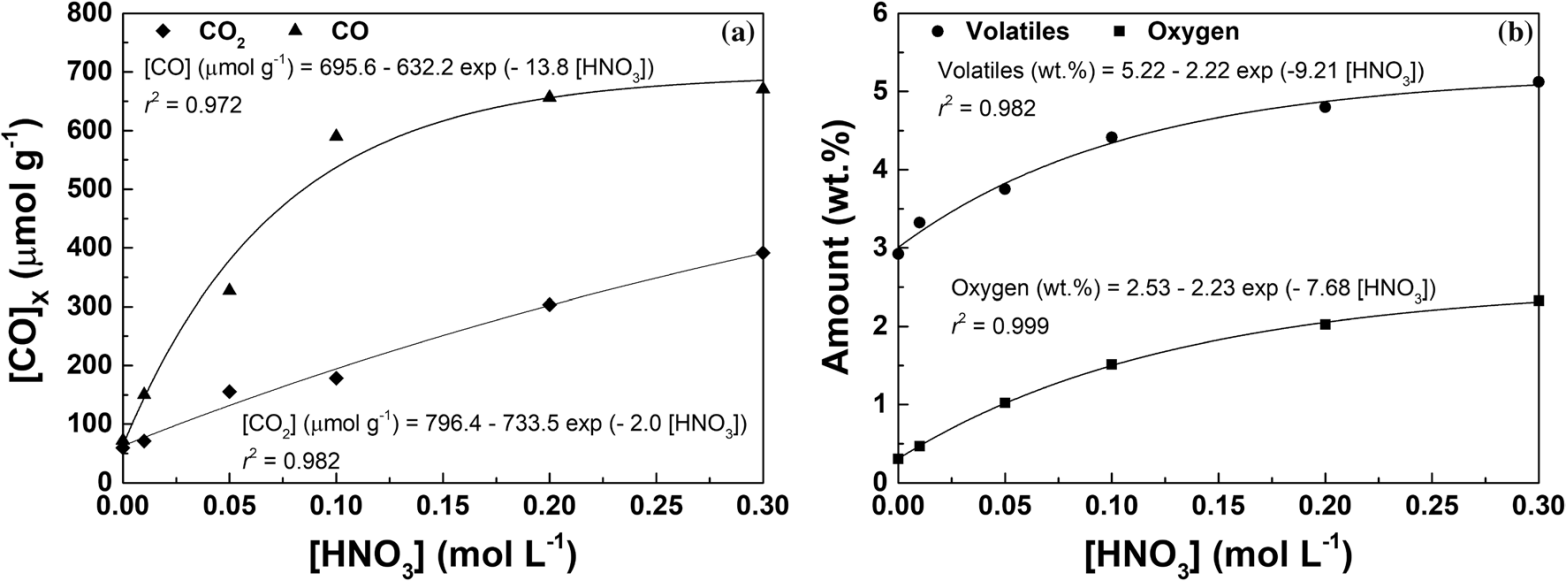
**(a)** Amounts of CO_2_ (*SD* ≤ 39 μmol g^−1^) and CO (*SD* ≤ 48 μmol g^−1^) released by TPD and **(b)** contents of oxygen (*SD* ≤ 0.19 wt%) and volatiles (*SD* ≤ 0.49 wt%) as function of the concentration of HNO_3_ employed in the hydrothermal treatment of MWCNTs. Points represent experimental data, while lines represent non-linear fittings.

The amounts of CO_2_ and CO increase as the concentration of the oxidizing agent increases (up to 0.30 mol L^−1^), confirming that MWCNTs are suitable to the inclusion of oxygenated functional groups through hydrothermal oxidation under mild conditions. The high level of oxidation in this type of carbon material can be associated to their structure, which provides a great number of defects where the oxidation process can be started^27^. Nevertheless, only a slight increase in the amount of oxygenated surface groups is observed when the HNO_3_ concentration is incremented from 0.20 to 0.30 mol L^−1^ (Fig. 3a,b; Table 1). This phenomenon was already reported in a previous work on HNO_3_ hydrothermal oxidation of carbon xerogels^23^, and suggests that there is a maximum extent of surface functionalization achievable through this mild hydrothermal methodology. A prevalence of surface groups released as CO was found in contrast to those released as CO_2_, the [CO]/[CO_2_] ratio being higher than one for all the MWCNTs under study (Table 1). The amount of oxygen follows the same trend of the surface groups released as CO_2_ and CO, as expected. Comparing the pristine MWCNTs and those hydrothermally treated with water (blank), both have similar (and low) amounts of oxygen surface groups, indicating that the hydrothermal treatment without addition of HNO_3_ has no effect on the surface chemistry of MWCNTs.

Correlations between the amount of oxygenated groups introduced on the surface of the MWCNTs (released as CO_2_ and CO) and the concentration of HNO_3_ employed in the hydrothermal treatment were obtained (Fig. 3a). Likewise, the contents of oxygen and volatiles (determined by TGA under inert atmosphere; Table 1) are also given as function of the HNO_3_ concentration (Fig. 3b). As observed, the evolution of all the parameters under study can be described as function of the HNO_3_ concentration by single exponential functions (*r*^2^ in the range 0.972–0.999), which is in accordance with our previous results on hydrothermally treated MWCNTs^24^, single-walled carbon nanotubes (SWCNTs)^25^ and carbon xerogels^23–25^. These correlations are very useful to fine tune the surface chemistry of MWCNTs, as they allow a given amount of oxygenated surface groups to be obtained by setting the proper concentration of HNO_3_ in the hydrothermal treatment.

**Figure 4:**
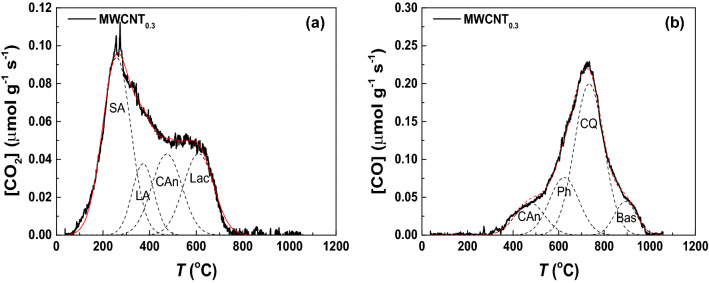
Deconvolution results of **(a)** CO_2_ and **(b)** CO TPD spectra of MWCNTs subjected to hydrothermal treatment with 0.30 mol L^−1^ HNO_3_ (MWCNT_0.3_). Dashed lines represent peaks assigned to strongly acidic carboxylic acids (SA), less acidic carboxylic acids (LA), carboxylic anhydrides (CAn), lactones (Lac), phenols (Ph), carbonyls and quinones (CQ) and basic surface groups (Bas), such as pyrones and chromenes. Red lines represent cumulative peak fitting.

Deconvolution analysis of the CO_2_ and CO TPD spectra was performed in order to identify and quantify the amounts of the different functionalities (Fig. 4a,b, respectively, and Figure S4, and corresponding results detailed in Tables S6 and S7). As a representative example, Fig. 4a,b show the deconvoluted CO_2_ and CO spectra of MWCTN_0.3_ (i.e., the material obtained after hydrothermal treatment with 0.30 mol L^−1^ HNO_3_). As observed, the surface groups released as CO_2_ were mainly assigned to strongly acidic carboxylic acids (SA, 172 µmol g^−1^), followed by carboxylic anhydrides (CAn, 84 µmol g^−1^) and lactones (Lac, 84 µmol g^−1^), and less acidic carboxylic acids (LA, 53 µmol g^−1^). Regarding the CO spectrum, the main contribution was assigned to carbonyls and quinones (CQ, 388 µmol g^−1^), followed by phenols (Ph, 148 µmol g^−1^) and carboxylic anhydrides (CAn, 84 µmol g^−1^). Moreover, a minor contribution was found at high temperature, as revealed by the shoulder observed at around 900 °C (Fig. 4b), which can be attributed to basic surface groups (Bas, 64 µmol g^−1^)^29^. Considering the results shown in Tables S6 and S7, the concentration of the oxygen functional groups generally increases with the concentration of HNO_3_ employed in the hydrothermal treatment, as previously observed for the total amounts of CO_2_ and CO.

The effect of the hydrothermal treatment on the overall surface charge (assessed though pH_PZC_ measurements) of the resulting materials, was also studied (Table 1). As observed, materials with a more pronounced acidic character are gradually obtained as the HNO_3_ concentration increases (i.e., pH_PZC_ is 6.9 for pristine MWCNTs and 5.0 for the MWCNTs treated with 0.30 mol L^−1^ HNO_3_). This continuous decrease of the pH_PZC_ values can be ascribed to the increasingly significant amounts of carboxilic acids (CA) introduced on the carbon surface, as summarized in Table 1. A similar conclusion was achieved in a previous publication of our group, upon a thorough analysis of results obtained by Raman spectroscopy^31^. As shown in Figure S5, the intensity ratio of the D band relative to the G mode (ID/IG) obtained by Raman spectroscopy plotted as a function of the total amount of functional groups determined by TPD revealed a linear function when characterizing single-walled carbon nanotubes treated with different HNO_3_ concentrations. Thus, data obtained by TPD correlate well with data obtained by Raman spectroscopy or other techniques such as water adsorption/desorption^31^.

The effect of the hydrothermal treatment on the textural properties of the MWCNTs was evaluated through N_2_ adsorption–desorption isotherms. All the MWCNTs possess negligible microporosity (as revealed by the low adsorption obtained at low N_2_ pressures); on the contrary, the prevalence of mesopores is revealed by the high adsorption observed at higher N_2_ pressures (Figure S6). The mesoporous nature of the MWCNTs is confirmed by the results given in Table 1. As observed, the surface area (both *S*_BET_ and *S*_meso_) and pore volume (*V*_pore_) increase as the concentration of HNO_3_ employed in the hydrothermal treatment increases. The average diameter of the MWCNTs is 9.5 nm (technical description provided by the manufacturer) and the average internal diameter of the tubes is around 4 nm^31^, i.e., almost 2-fold higher than the maximum diameter of micropores (2 nm). Therefore, as expected, micropores were not found in the original sample (*V*_micro_ = 0) and the values were very low for the oxidized ones (i.e., within the error of the analysis).

**Figure 5:**
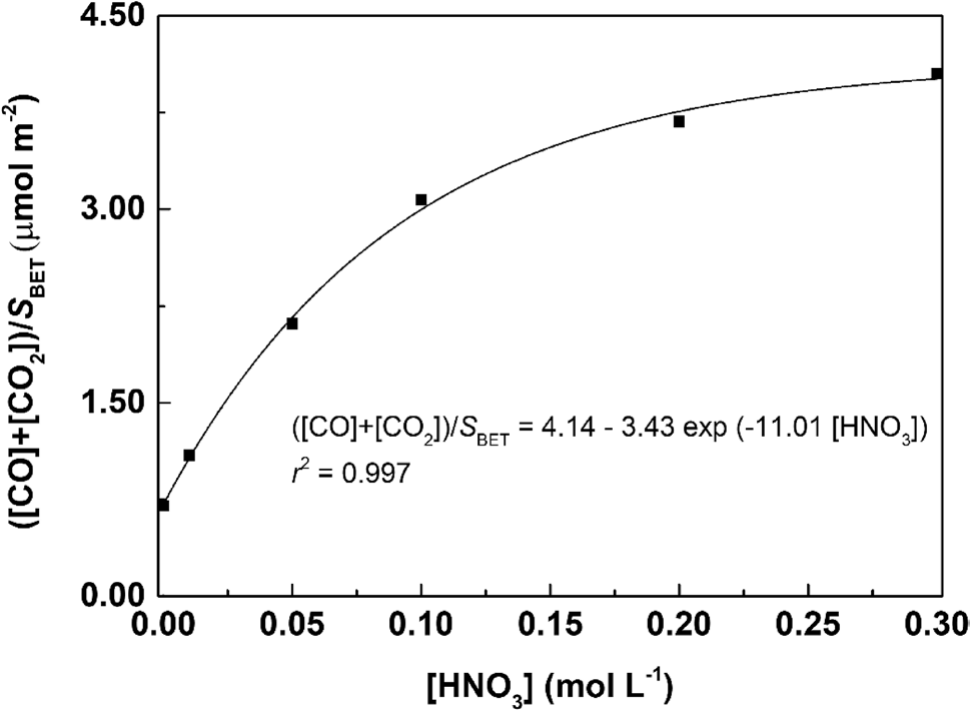
([CO_2_] + [CO])/*S*_BET_ as a function of HNO_3_ concentration.

Our results are in line with those previously reported in a study performed with SWCNTs, in which it was concluded that functionalization with HNO_3_ causes the opening of the nanotube caps with no significant defects being additionally produced^32^. The progressive increase of *S*_BET_, *S*_meso_ and *V*_pore_ can thus be ascribed to the opening of the nanotube caps, which enhances the accessibility to the inner part of the MWCNTs, rather than defect creation. As stated in a previous work, a gradual increase of both *S*_BET_ and the amount of oxygenated functional groups is observed when the hydrothermal oxidation treatment is performed with increasing HNO_3_ concentrations^24^. This methodology leads to a slight increase of the *S*_BET_, around 32% when comparing the pristine and the MWCNT_0.3_ sample, i.e. from 198 m^2^ g^−1^ in the pristine MWCNTs to 262 m^2^ g^−1^ in the sample treated with 0.30 mol L^−1^ HNO_3_. This increase of *S*_BET_ as consequence of the hydrothermal oxidation treatment with HNO_3_ is similar to that obtained in a previous study of our group performed under similar conditions, i.e., 27%^33^. In that study, both pristine and oxidized (0.3 mol L^−1^ of HNO_3_) MWCNTs were characterized by scanning electron microscopy (SEM) (Figure S7), allowing to conclude that both samples consist of agglomerated carbon nanotubes, with no significant morphological changes being perceptible as a consequence of the HNO_3_ hydrothermal oxidation^33^. When the total amounts of CO_2_ and CO released by TPD are normalized by the *S*_BET_ (i.e., ([CO_2_] + [CO])/*S*_BET_), and represented as a function of HNO_3_ concentration (Fig. 5), two distinct stages in the exponential curve are observed: (i) in the first stage, the pronounced increase is associated with the functionalization of the accessible surface area of the original MWCNTs; while (ii) the final part of the curve corresponds to the functionalization of new surface area made accessible during the HNO_3_ hydrothermal oxidation. The correlation obtained in Fig. 5 (with *r*^2^ = 0.997) can be extended to other carbon materials obtained through the same hydrothermal functionalization methodology.

### Application of functionalized MWCNTs for extraction of EU multi-class OMPs

**Figure 6:**
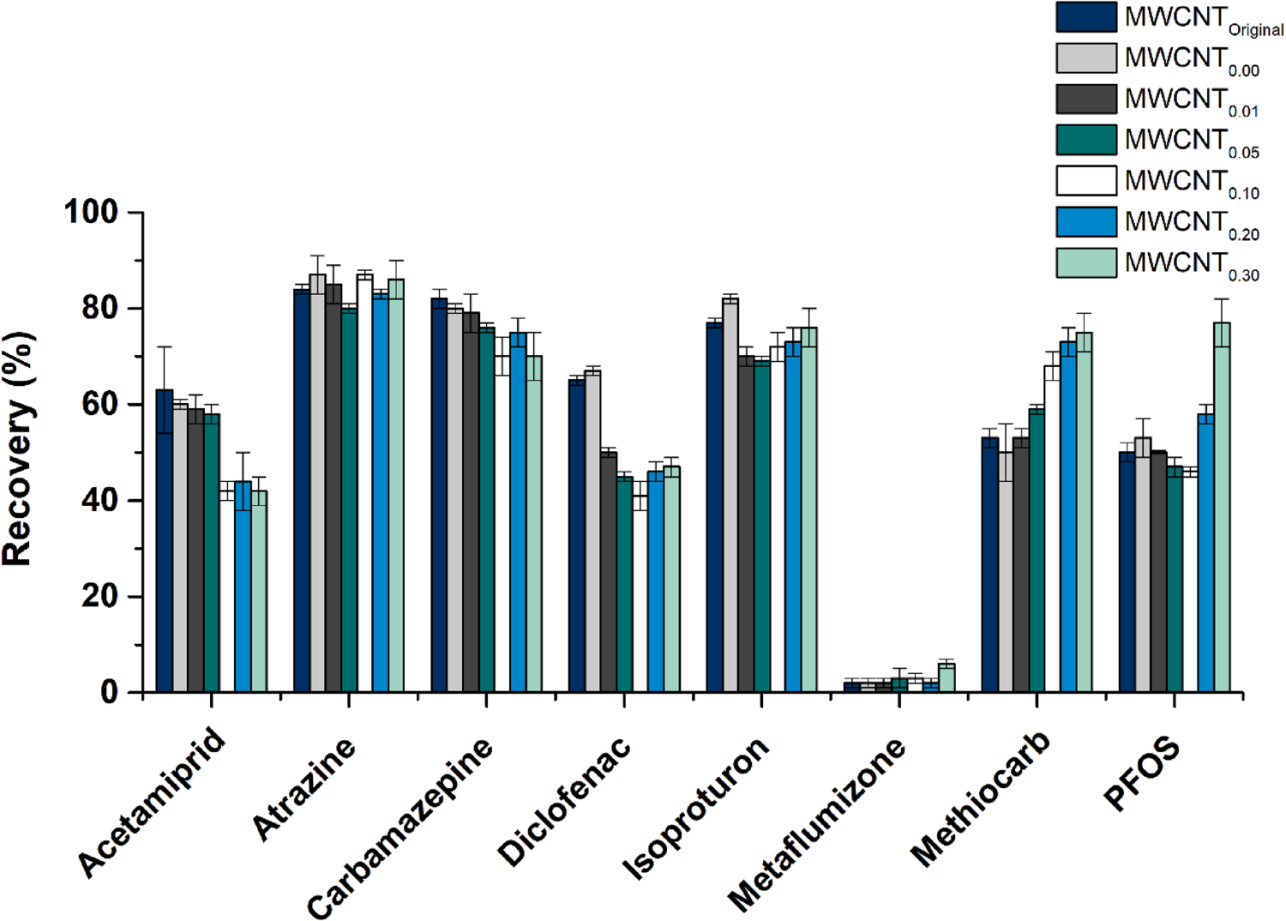
Recoveries obtained for the target micropollutants (200 ng L^−1^ each), when using cartridges packed with MWCNTs (50 mg) obtained after hydrothermal treatment with different HNO_3_ concentrations (0–0.30 mol L^−1^). Experiments performed with 500 mL of sample (SW; pH 3) and using ethanol as solvent (4 mL); n = 3 (RSD is represented as error bars).

The applicability of the original and hydrothermally treated MWCNTs as sorbents for SPE of the 8 target OMPs was studied, as well as the influence of both textural and surface chemistry properties of these materials in the adsorption/desorption process. For that purpose, the recoveries of the target compounds were determined as described by Eq. (1) (“Evaluation of the SPE recovery efficiency” section). The recoveries obtained revealed different behaviors for the OMPs under study (Fig. 6) due to their distinct classes and physicochemical properties. For instance, similar recoveries were achieved for the pesticides atrazine and isoproturon (around 80 and 70%, respectively) with all the samples of MWCNTs that were tested, indicating that the adsorption/desorption process is not affected by the oxygenated surface groups introduced by the HNO_3_ hydrothermal treatment. The SPE cartridges packed with the original MWCNTs (recoveries > 60%) performed better than the materials treated with HNO_3_ for the pharmaceutical compounds diclofenac and carbamazepine and the neonicotinoid pesticide acetamiprid (recoveries < 60%). In the case of metaflumizone, the recovery obtained is ineffective with the original and treated MWCNTs packed in the SPE cartridges. On the other hand, performing SPE with MWCNT_0.30_ leads to a significant improvement of the recoveries obtained for methiocarb and the industrial compound PFOS, when compared to the original MWCNTs.

Several mechanisms may simultaneously control the adsorption/desorption process of the organic pollutants on MWCNTs, including (i) π–π interactions, i.e., the interactions between bulk π systems present on the surface of MWCNTs and organic molecules with their benzene rings or C=C double bonds; (ii) hydrogen bonds with functional groups on the surface of the sorbent material; and (iii) electrostatic interactions due to of the charged carbon material surface^10,34^. However, each mechanism could be affected differently by the environmental conditions, which makes the application of MWCNTs for SPE of different organic compounds from aqueous matrices a challenging research topic. The obtained results (Fig. 6) suggest that the HNO_3_ hydrothermal treatment applied to MWCNTs affects the SPE efficiency of the target OMPs in two distinct ways. In the case of acetamiprid, diclofenac and carbamazepine, the dominant adsorption mechanism seems to be π–π dispersive interactions, which decrease with the increase of the oxygen-containing functional groups, most of them with electron-withdrawing properties. In contrast, for methiocarb and PFOS, the HNO_3_ functionalization leads to higher recoveries, possibly due to the predominance of the hydrogen bond adsorption mechanism favoured by the increase of the oxygen surface groups.

**Figure 7:**
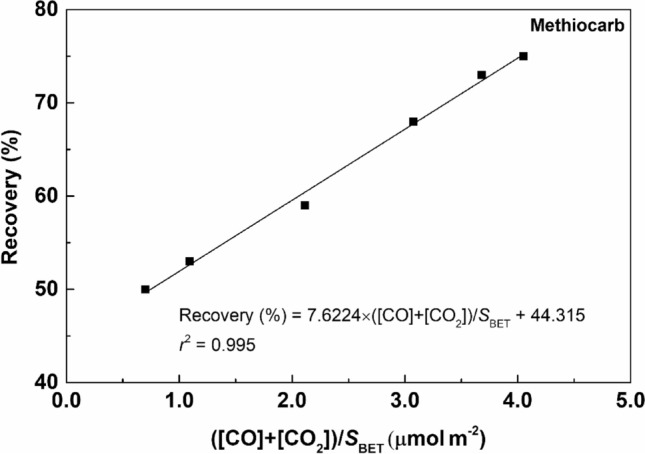
Recovery obtained for methiocarb as a function of ([CO_2_] + [CO])/*S*_BET_.

In the case of the carbamate pesticide methiocarb, it is interesting to observe the continuous increase in the recovery values with the increase of the acid concentration. Thus, the recoveries obtained for methiocarb were plotted as a function of the total amount of functional groups introduced (CO + CO_2_) divided by the respective *S*_BET_ (Fig. 7). A good linear correlation (*r*^*2*^ = 0.995) was obtained, i.e., the total amount of CO and CO_2_ divided by the S_BET_ has proved to be a good predictor of methiocarb recovery. For this carbamate pesticide, the HNO_3_ functionalization of MWCNTs led to a continuous and significant increase in the SPE efficiency. In order to understand if this correlation was associated with any specific functional group previously determined by TPD, a similar analysis was made but with the amount of each surface group (SA, LA, CAn, Lac, Ph, CQ and Bas), instead of the total amount released as CO and CO_2_. Good correlations with Ph (*r*^*2*^ = 0.916) and CQ groups (*r*^*2*^ = 0.918) were also obtained (Figure S8a and b), suggesting that the presence of higher amounts of these functional groups on the MWCNT surface increases the affinity for methiocarb.

In the literature, there are several studies reporting the possible mechanisms of adsorption of some target compounds on MWCNTs. For example, in the case of atrazine, π–π dispersive and polar interactions were appointed as responsible for the adsorption on MWCNTs^35^. Regarding the industrial compound PFOS, a study conducted by Li, et al.^36^ concluded that hydrophobic interactions are the main mechanism of adsorption of PFOS. The pharmaceutical compound diclofenac was already studied, and diverse types of interactions were suggested, such as electrostatic and hydrophobic interactions and hydrogen bonding^34,37^. In the case of carbamazepine, the π–π electron–donor–acceptor interactions, hydrogen bonding and hydrophobic interactions had a key role in the adsorption on MWCNTs^34,37^. Therefore, the mechanism and the extraction performance result from the interplay of the characteristics of each pollutant and the properties of the sorbent material.

## Conclusions

Pristine and modified MWCNTs were applied as adsorbent materials in conventional SPE for enrichment of 8 EU multi-class OMPs in SW samples and analysis by UHPLC-MS/MS. The optimized SPE procedure with pristine MWCNTs has the great advantage of using an eco-friendly solvent (ethanol) for both conditioning and elution steps. Additional advantages of this carbon-based cartridge are the small amount of adsorbent that is needed (50 mg), representing a ~ 75% cost reduction in comparison with the commercial cartridge (while obtaining similar recoveries), and the ability to be reused at least three times without substantial impact on the retention capacity of the adsorbent. The oxidation of the MWCNTs surface (and thus the introduction of oxygenated functional groups) can affect the SPE recoveries in different ways. The dominant adsorption mechanism seems to be π–π dispersive interactions in the case of acetamiprid, diclofenac and carbamazepine (i.e. the recoveries were higher when using the original MWCNTs), whereas the hydrogen bond adsorption mechanism (favoured by the increase of the oxygen surface groups) seems to be predominant in the case of methiocarb and PFOS. Moreover, a very good correlation between the recovery of methiocarb and the functionalities created on the MWCNTs was found, which was attributed to the phenol and carbonyl and quionone groups. The fine control of the surface chemistry and texture of MWCNTs, with the purpose of improving the selectivity and specificity of these materials, opens a window of opportunity for the development of more efficient and eco-friendly analytical tools for the analysis of EU-relevant OMPs, for instance by mixing MWCNTs with different textural and surface chemistry properties in the same SPE cartridge.

## Supplementary Information

Supplementary Information.
