# Supplementary material for: Solid-phase extraction cartridges with multi-walled carbon nanotubes and effect of the oxygen functionalities on the recovery efficiency of organic micropollutants
Source: Sci Rep. 2020 Dec 18;10:22304. doi: 10.1038/s41598-020-79244-8 (PMC7749141; doi:10.1038/s41598-020-79244-8)
Supplement: Supplementary file 1 — Supplementary Information. [file 41598_2020_79244_MOESM1_ESM.docx]

**Supplementary Material**

**Solid-phase extraction cartridges with multi-walled carbon nanotubes and effect of the oxygen functionalities on the recovery efficiency of organic micropollutants**

Marta O. Barbosa, Rui S. Ribeiro, Ana R.L. Ribeiro, M. Fernando R. Pereira, Adrián M.T. Silva*

Laboratory of Separation and Reaction Engineering - Laboratory of Catalysis and Materials (LSRE-LCM), Faculdade de Engenharia, Universidade do Porto, Rua Dr. Roberto Frias s/n, 4200-465 Porto, Portugal.

*Corresponding author: adrian@fe.up.pt (Adrián M.T. Silva)

**Supplementary Material Table of Contents**

**Text S1.** Reference SPE protocol.

**Text S2.** MWCNTs SPE optimization.

**Table S1.** Target compounds, class, structure, relative molecular mass (M_r_), p*K*a and log K_OW_ values and solubility in water.

**Table S2.** Selected reaction monitoring (SRM) instrument parameters for tandem mass spectrometry analysis of target analytes.

**Table S3.** Retention time, range, linearity, instrument and method detection and quantification limits for each target analyte.

**Table S4.** Textural properties of MWCNTs (NC3100) and Oasis HLB: specific surface area (*S*_BET_), non-microporous specific surface area (*S*_meso_), total pore volume (*V*_total_) and average pore diameter (*d*_pore_).

**Table S5.** Studies dealing with the application of MWCNTs in conventional SPE: target analyte and respective spiked level (ng L^-1^), matrix, sample loading (mL), amount of MWCNTs packed in the cartridge (mg), conditioning and elution solvents, and recoveries (%) obtained. Pollutants included in these studies that are out of the scope of EU legislation are not discussed.

**Table S6.** Results obtained from the deconvolution of the CO_2_ spectra of MWCNTs subjected to hydrothermal treatment with different HNO_3_ concentrations. *T*_M_, *W* and *A* represent the temperature at the peak maximum, the width of the peak at half-height and the integrated peak area, respectively. Peaks were assigned to strongly acidic carboxylic acids (SA), less acidic carboxylic acids (LA), carboxylic anhydrides (Can) and lactones (Lac).

**Table S7.** Results obtained from the deconvolution of the CO spectra of MWCNTs subjected to hydrothermal treatment with different HNO_3_ concentrations. *T*_M_, *W* and *A* represent the temperature at the peak maximum, the width of the peak at half-height and the integrated peak area, respectively. Peaks were assigned to carboxylic anhydrides (Can), phenols (Ph), carbonyls and quinones (CQ) and basic surface groups (Bas), such as pyrones and chromenes.

**Figure S1.** Schematic representation (a) and photograph (b) of the lab-scale packing device designed to prepare MWCNT cartridges (6 at a time).

**Figure S2.** Schematic representation of the experimental procedure carried out to evaluate the extraction efficiency (i.e. recovery in %) of each SPE method.

**Figure S3.** Total Ion Current (TIC) chromatograms of the 8 target OMPs (200 ng L^-1^) in: (a) a SPE extract of a spiked sample; and (b) a post-spiked blank extract, using cartridges packed with MWCNTs (50 mg).

**Figure S4.** Deconvolution results of (a, c, e, g, i) CO_2_ and (b, d, f, h, j) CO TPD spectra of MWCNTs subjected to hydrothermal treatment with different HNO_3_ concentrations. Dashed lines represent peaks assigned to strongly acidic carboxylic acids (SA), less acidic carboxylic acids (LA), carboxylic anhydrides (Can), lactones (Lac), phenols (Ph), carbonyls and quinones (CQ) and basic surface groups (Bas), such as pyrones and chromenes. Red lines represent cumulative peak fitting.

**Figure S5.** Intensity ratio of the D band relative to the G mode (*I*_D_/*I*_G_) obtained by Raman spectroscopy, as function of the total amount of evolved CO and CO_2_ determined by TPD when functionalizing single-walled carbon nanotubes with different HNO_3_ concentrations in the hydrothermal treatment. Lines designate the linear fit. Reprinted (adapted) with permission from The Journal of Physical Chemistry C, Vol. 115, G.E. Romanos, V. Likodimos, R.R.N. Marques, T.A. Steriotis, S.K. Papageorgiou, J.L. Faria, J.L. Figueiredo, A.M.T. Silva, P. Falaras, Controlling and Quantifying Oxygen Functionalities on Hydrothermally and Thermally Treated Single-Wall Carbon Nanotubes, 8534-8546. Copyright 2011, with permission of American Chemical Society.

**Figure S6.** N_2_ adsorption-desorption isotherms at -196 ^o^C of MWCNTs subjected to hydrothermal treatment with different HNO_3_ concentrations.

**Figure S7.** SEM micrographs of (a) pristine (MWp) and (b) functionalized (MWf) MWCNTs. Reprinted from The Journal of Membrane Science, Vol. 520, Sergio Morales-Torres, Carla M.P. Esteves, José L.Figueiredo, Adrián M.T. Silva, Thin-film composite forward osmosis membranes based on polysulfone supports blended with nanostructured carbon materials, 326-336, Copyright 2016, with permission from Elsevier [License number: 4945340204301].

**Figure S8.** Recovery obtained for methiocarb as a function of (a) (Ph)/S_BET_ and (b) (CQ)/S_BET_.

**Text S1. Reference SPE protocol.**

An offline SPE method with commercial Oasis HLB cartridges was used as reference protocol, according to our previous works [1, 2]. Briefly, Oasis HLB cartridges were successively conditioned with 4 mL of ethanol and 4 mL of ultrapure water at a flow rate of 1 mL min^−1^. Sample loading was carried out with 500 mL of acidified (pH 3) blank and spiked (200 ng L^-1^ of each target compound) SW samples at a constant flow rate of 10 mL min^-1^, using a vacuum manifold unit connected to a vacuum pump. The washing step was performed with 4 mL of ultrapure water. The cartridges were then dried under vacuum for 45 min. The elution step was performed at a flow rate of 1 mL min^−1^ with 4 mL of ethanol and the extracts were evaporated to dryness in a Centrivap Concentrator device (LABCONCO Corporation, Kansas City, MO, USA). The dried extracts were reconstituted in 250 μL of ethanol and the resulting ethanolic extracts were filtered through 0.22 μm polytetrafluoroethylene syringe filters (Membrane Solutions, Kent, WA, USA) to be injected into the UHPLC-MS/MS system.

**Text S2. MWCNTs SPE optimization.**

In order to study the performance of functionalized MWCNTs as SPE adsorbents for enrichment of the target OMPs, the main experimental conditions affecting the extraction efficiency of pristine MWCNTs (NC3100) were optimized in detail. All experiments carried out during the optimization were performed in triplicate. The pristine MWCNTs cartridges were prepared manually, using a lab-scale packing device specifically designed for that purpose (Figure S1). This procedure involved four sequential steps: (i) one polyethylene frit (20 µm) was positioned on the bottom of an empty cartridge (6 mL); (ii) a certain amount (25, 50, 75, 100 or 150 mg) of pristine MWCNTs (NC3100) was then introduced; (iii) the sample was covered with another polyethylene frit; and (iv) slightly compressed until a specific bed height was reached. Cartridges packed with 150 mg of MWCNTs were used in preliminary studies to assess the effect of different sample pH in SPE. The SW samples (500 mL) were adjusted to different pH (3 and 9) and extracted using ethanol, methanol or acetonitrile (4 mL) and then ultrapure water (4 mL) was passed through the SPE cartridge at a flow rate of 1 mL min^−1^. A sample pH of 7 was also tested, using ethanol as extraction solvent. After selecting the best sample pH and extraction solvent, the effect of the amount of the adsorbent material packed in the SPE cartridge was studied in the range 25 – 150 mg. Sample loading was carried out with 50, 100, 250, 500 or 1000 mL of blank and spiked SW samples (200 ng L^-1^ of each target compound). After washing and drying, the retained analytes were eluted with 4, 6, 8 or 10 mL of ethanol, as this volume can also influence the enrichment efficiency and the overall cost of the SPE method. The elution volume should be enough to elute the analytes from the sorbent material; however, excessive amounts represent waste and avoidable costs. The resulting extracts were reconstituted and filtered as the reference protocol.

**Table S1.** Target compounds, class, structure, relative molecular mass (M_r_), p*K*a and log *K*_OW_ values and solubility in water.

| **Compound** | **Class and sub class** | **Structure** | **M_r_** | **p*K*a** | **log *K*_OW_** | | **Solubility in water (mg L^-1^)** | |  |
| --- | --- | --- | --- | --- | --- | --- | --- | --- | --- |
| Acetamiprid^a^ | Pesticide  *Neonicotinoid* | 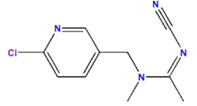 | 222.68 | 0.70 | | 0.80 | | 4250  (25 ºC) | |
| Atrazine^b^ | Pesticide  *Triazine* | 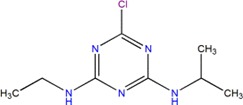 | 215.69 | 1.60 | | 2.61 | | 33.0  (25 ºC) | |
| Carbamazepine | Pharmaceutical  *Psychiatric drug* | 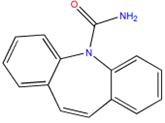 | 236.27 | 13.9 | | 2.45 | | 17.7  (25 ºC) | |
| Diclofenac^c^ | Pharmaceutical  *Anti-inflammatory* | 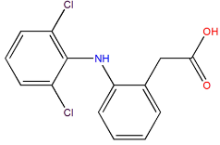 | 296.14 | 4.15 | | 4.51 | | 2.5  (25 ºC) | |
| Isoproturon^b^ | Pesticide  *Phenylurea* | 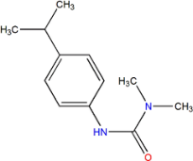 | 206.28 | n.a. | | 2.87 | | 70.0  (20 ºC) | |
| Metaflumizone^a^ | Pesticide  *Insecticide* | 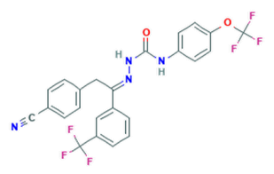 | 506.41 | n.a | | n.a | | n.a | |
| Methiocarb^a^ | Pesticide  *Insecticide* | 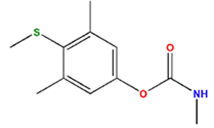 | 225.31 | 14.8 | | 2.92 | | 27.0  (20 ºC) | |
| Perfluorooctanesulfonic acid (PFOS)^b^ | Industrial compound | 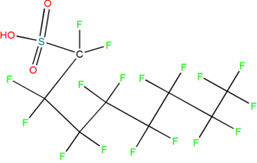 | 500.13 | -3.30 | | 4.49 | | 3.2x10^-3^ (25 ºC) | |

^a^ Contaminant of emerging concern of Decision 2018/840/EU;

^b^ Priority substance of Directive 2013/39/EU;

^c^ Contaminant of emerging concern of the former Decision 2015/495/EU;

n.a. - not available.

**Table S2.** Selected reaction monitoring (SRM) instrument parameters for tandem mass spectrometry analysis of target analytes.

| **Analyte** | **IS set^a^** | **ESI mode (NI^b^ or PI^c^)** | **Precursor ion (m/z)** | **Quantification (SRM1)** | | | | **Confirmation (SRM2)** | | | |
| --- | --- | --- | --- | --- | --- | --- | --- | --- | --- | --- | --- |
|  |  |  |  | **Product Ion** | **DP^d^ (V)** | **CE^e^ (V)** | **CXP^f^ (V)** | **Product Ion** | **DP^d^ (V)** | **CE^e^ (V)** | **CXP^f^ (V)** |
| Acetamiprid | 1 | PI | 222.70 | 126.00 | -15 | -20 | -23 | 56.10 | -15 | -16 | -22 |
| Acetamiprid-d3 (1) | - | PI | 226.00 | 126.00 | -25 | -20 | -23 | - | - | - | - |
| Atrazine | 2 | PI | 216.00 | 174.00 | -24.0 | -16.0 | -30.0 | 68.05 | -24.0 | -36.0 | -10.0 |
| Atrazine-d5 (2) | - | PI | 221.10 | 179.05 | -11.0 | -18.0 | -17.0 | - | - | - | - |
| Carbamazepine | 4 | PI | 237.00 | 194.00 | -12.0 | -20.0 | -30.0 | 192.00 | -12.0 | -25.0 | -30.0 |
| Diclofenac | 3 | NI | 293.90 | 250.00 | 21.0 | 11.0 | 17.0 | 214.05 | 21.0 | 20.0 | 22.0 |
| Diclofenac-d4 (3) | - | NI | 297.95 | 254.05 | 21.0 | 12.0 | 28.0 | - | - | - | - |
| Fluoxetine-d5 (4) | - | PI | 315.10 | 44.00 | -15.0 | -20.0 | -15.0 | - | - | - | - |
| Isoproturon | 2 | PI | 206.80 | 72.00 | -15.0 | -20.0 | -29.0 | 46.00 | -15.0 | -18.0 | -16.0 |
| Metaflumizone | 3 | NI | 505.00 | 302.05 | 36.0 | 18.0 | 21.0 | 116.95 | 36.0 | 43.0 | 22.0 |
| Methiocarb | 5 | PI | 226.10 | 169.10 | -24.0 | -9.0 | -17.0 | 121.10 | -24.0 | -19.0 | -21.0 |
| Methiocarb-d3 (5) | - | PI | 229.10 | 169.10 | -25.0 | -11.0 | -30.0 | - | - | - | - |
| Perfluorooctanesulfonic acid | 2 | NI | 498.70 | 79.95 | 18.0 | 50.0 | 14.0 | 99.00 | 18.0 | 46.0 | 18.0 |

^a^ IS is internal standard;

^b^ NI is negative ionization mode;

^c^ PI is positive ionization mode;

^d^ DP is the declustering potential;

^e^ CE is the collision energy;

^f^ CXP is the collision cell exit potential.

**Table S3.** Retention time, range, linearity, instrument and method detection and quantification limits for each target analyte.

| **Analyte** | **Retention time**  **(min)** | **Range**  **(ng L^-1^)** | ***r^2^*** | **IDL^a^**  **(µg L^-1^)** | **IQL^b^**  **(µg L^-1^)** | **MDL^c^**  **(ng L^-1^)** | **MQL^d^**  **(ng L^-1^)** |
| --- | --- | --- | --- | --- | --- | --- | --- |
| Acetamiprid | 1.19 | 3.06 – 200 | 0.999 | 16.1 | 48.7 | 1.01 | 3.06 |
| Atrazine | 1.83 | 4.61 – 200 | 0.993 | 8.79 | 26.6 | 1.52 | 4.61 |
| Carbamazepine | 1.52 | 2.09 – 200 | 0.999 | 1.61 | 4.90 | 0.69 | 2.09 |
| Diclofenac | 3.86 | 3.74 – 200 | 0.998 | 3.62 | 11.3 | 1.23 | 3.74 |
| Isoproturon | 2.07 | 11.7 – 200 | 0.999 | 9.24 | 28.0 | 3.85 | 11.7 |
| Metaflumizone | 24.5 | 1.35 – 200 | 0.999 | 2.02 | 6.13 | 0.45 | 1.35 |
| Methiocarb | 2.68 | 2.69 – 200 | 0.997 | 0.74 | 2.24 | 0.89 | 2.69 |
| Perfluorooctanesulfonic acid | 1.47 | 11.7 – 200 | 0.997 | 25.4 | 77.1 | 3.87 | 11.7 |

^a^ IDL is instrument detection limit;

^b^ IQL is instrument quantification limit;

^c^ MDL is method detection limit;

^d^ MQL is method quantification limit.

**Table S4.** Textural properties of MWCNTs (NC3100) and Oasis HLB: specific surface area (*S*_BET_), non-microporous specific surface area (*S*_meso_), total pore volume (*V*_total_) and average pore diameter (*d*_pore_).

| **Adsorbent used in the**  **SPE cartridge** | **Parameters** | | | |  |
| --- | --- | --- | --- | --- | --- |
|  | ***S*_BET_**  **(m^2^ g^-1^)** | ***S*_meso_**  **(m^2^ g^-1^)** | ***V*_total_**  **(cm^3^ g^-1^)** | ***d*_pore_**  **(nm)** | |
| MWCNTs (NC3100) | 198 | 155 | 1.264 | 25.5 | |
| Oasis HLB | 756 | 607 | 1.284 | 6.8 | |

Table S5. Studies dealing with the application of MWCNTs in conventional SPE: target analyte and respective spiked level (ng L^-1^), matrix, sample loading (mL), amount of MWCNTs packed in the cartridge (mg), conditioning and elution solvents, and recoveries (%) obtained. Pollutants included in these studies that are out of the scope of EU legislation are not discussed.

| **Target analyte**  **(spiked level)** | **Matrix** | **Sample loading (mL)** | **Amount of MWCNTs (mg)** | **Conditioning and elution solvents** | **Recovery**  **(%)** | | **Ref.** |
| --- | --- | --- | --- | --- | --- | --- | --- |
| Atrazine and simazine  (100 ng L^-1^) | Tap and groundwater | 700 | 300 | Conditioning: 10 mL ACN + 10 mL of triply distilled water; Elution: 8 mL MeOH | 85–95 | [3] | |
| Atrazine (300, 1000 and 5000 ng L^-1^) | Surface and underground water | 1000 | 500 | Conditioning: 3 mL MeOH + 5 mL water; Elution: 4 mL ethyl acetate | 86–110 | [4] | |
| Atrazine and simazine (20000 ng L^-1^) | River water | 500 | 100 | Conditioning: 5 mL ACN + 3 mL aqueous solution (pH 3); Elution: 5 mL mixed solution of 90% ACN and 10% H_2_O | 87–97 | [5] | |
| Atrazine and simazine  (800 ng L^-1^) | River, tap, reservoir and wastewater | 500 | 100 | Conditioning: 5 mL ACN + 5 mL water; Elution: 4 mL ACN | 83–104 | [6] | |
| Atrazine  (50000 ng L^-1^) | Tap, reservoir and stream  water | 50 | 200 | Conditioning: 5 mL ACN + 5 mL water; Elution: 5 mL ACN | 81–108 | [7] | |
| Chlorpyrifos (100, 300 and 800 ng L^-1^) | Mineral water, groundwater, and agricultural run-off water | 800 | 40 | Conditioning: 10 mL ACN + 10 mL Milli-Q water; Elution: 20 mL DCM | 70–100 | [8] | |
| Chlorpyrifos (1000000, 5000000 and  10000000 ng L^-1^) | Well, tap and river water | 500 | 100 | Conditioning: 5 mL DCM + 5 mL water; Elution: 3 mL DCM | 94–98 | [9] | |
| Atrazine  (500 ng L^-1^) | Tap and reservoir water | 600 | 300 | Conditioning: 10 mL ACN + 10 mL of triply distilled water; Elution: 8 mL MeOH | 74–>99 | [10] | |
| Methiocarb  (5000 ng L^-1^) | Tap and SW | 200 | 100 | Conditioning: 5 mL MeOH + 5 mL Milli-Q water; Elution: 8 mL dichloromethane | 92–96 | [11] | |
| Alachlor  (1000 ng L^-1^) | Tap and river water | 500 | 100 | Conditioning: 2 mL ethyl–acetate + 2 mL MeOH + 2 mL of distilled water; Elution: 7 mL ethyl–acetate | 82–85 | [12] | |
| Thiamethoxam, acetamiprid and imidacloprid  (800 ng L^-1^) | Tap, ground and  reservoir water | 200 | 100 | Conditioning: EtOH + Milli-Q water; Elution: 4 mL MeOH | 88–110 | [6] | |
| PFOS  (500 ng L^-1^) | Tap and river water | 500 | 200 | Conditioning: 5 mL MeOH + 5 mL ultrapure water + 5 mL ultrapure water (pH 3) | 88–­90 | [13] | |
| Pentachlorophenol (PCP) (200000 ng L^-1^) | River water | 100 | 20 | Conditioning: 5 mL of deionized water + 5 mL MeOH + 5 mL of deionized water; Elution: 5 mL acetone | 62–98 | [14] | |
| PCP  (5000 ng L^-1^) | Tap and river water | 200 | 300 | Conditioning: 5 mL MeOH + 5 mL ultrapure water; Elution: 6 mL MeOH (pH 10) | 97–109 | [15] | |
| Polycyclic aromatic hydrocarbons (PAHs)  (200 ng L^-1^) | Tap, river and seawater | 500 | 150 | Conditioning: 10 mL n-hexane + 10 mL MeOH + 10 mL water; Elution: 15 mL n-hexane | 67–127 | [16] | |
| PAHs  (80 – 20000 ng L^-1^) | River, tap and wastewater | 500 | 500 | Conditioning: 5 mL MeOH + 5 mL water; Elution: 4 mL ACN | 79–118 | [17] | |
| Erythromycin, azithromycin and diclofenac  (1000 ng L^-1^) | Surface and groundwater | 100 | 50 | Conditioning: 5 mL MeOH-dichloromethane (1:1, v/v) + 5 mL deionized water; Elution: 15 mL MeOH-dichloromethane (1:1, v/v) | 93–112 | [18] | |
| Diclofenac  (50 and 100 ng L^-1^) | River water | 100 | 20 | Conditioning: 2 mL MeOH + 2 mL ultrapure water (pH 8); Elution: 7 mL MeOH containing 10% (v/v) of ammonium hydroxide (25% purity) | 79–94 | [19] | |

**Table S6.** Results obtained from the deconvolution of the CO_2_ spectra of MWCNTs subjected to hydrothermal treatment with different HNO_3_ concentrations. *T*_M_, *W* and *A* represent the temperature at the peak maximum, the width of the peak at half-height and the integrated peak area, respectively. Peaks were assigned to strongly acidic carboxylic acids (SA), less acidic carboxylic acids (LA), carboxylic anhydrides (Can) and lactones (Lac).

| **[HNO_3_]**  **(mol L^-1^)** | **Peak #1** | | |  | **Peak #2 (SA)** | | |  | **Peak #3 (LA)** | | |  | **Peak #4 (CAn)** | | |  | **Peak #5 (Lac)** | | |
| --- | --- | --- | --- | --- | --- | --- | --- | --- | --- | --- | --- | --- | --- | --- | --- | --- | --- | --- | --- |
|  | ***T*_M_ (^o^C)** | ***W* (^o^C)** | ***A***  **(µmol g^-1^)** |  | ***T*_M_ (^o^C)** | ***W* (^o^C)** | ***A***  **(µmol g^-1^)** |  | ***T*_M_ (^o^C)** | ***W* (^o^C)** | ***A***  **(µmol g^-1^)** |  | ***T*_M_ (^o^C)** | ***W* (^o^C)** | ***A***  **(µmol g^-1^)** |  | ***T*_M_ (^o^C)** | ***W* (^o^C)** | ***A***  **(µmol g^-1^)** |
| 0 (Blank) | - | - | 0 |  | - | 0 | 0 |  | 352 | 71 | 5 |  | 484 | 146 | 30 |  | 580 | 146 | 25 |
| 0.01 | 131 | 37 | 3 |  | 230 | 167 | 16 |  | 352 | 95 | 4 |  | 507 | 161 | 26 |  | 639 | 161 | 21 |
| 0.05 | 137 | 39 | 8 |  | 234 | 150 | 48 |  | 360 | 90 | 6 |  | 472 | 173 | 40 |  | 626 | 173 | 55 |
| 0.1 | - | - | 0 |  | 231 | 117 | 60 |  | 353 | 163 | 33 |  | 492 | 166 | 35 |  | 631 | 166 | 49 |
| 0.2 | - | - | 0 |  | 244 | 109 | 123 |  | 370 | 141 | 29 |  | 435 | 171 | 76 |  | 609 | 171 | 75 |
| 0.3 | - | - | 0 |  | 259 | 143 | 172 |  | 372 | 110 | 53 |  | 474 | 153 | 84 |  | 610 | 153 | 84 |

**Table S7.** Results obtained from the deconvolution of the CO spectra of MWCNTs subjected to hydrothermal treatment with different HNO_3_ concentrations. *T*_M_, *W* and *A* represent the temperature at the peak maximum, the width of the peak at half-height and the integrated peak area, respectively. Peaks were assigned to carboxylic anhydrides (Can), phenols (Ph), carbonyls and quinones (CQ) and basic surface groups (Bas), such as pyrones and chromenes.

| **[HNO_3_]**  **(mol L^-1^)** | **Peak #1** | | |  | **Peak #2 (CAn)** | | |  | **Peak #3 (Ph)** | | |  | **Peak #4 (CQ)** | | |  | **Peak #5 (Bas)** | | |
| --- | --- | --- | --- | --- | --- | --- | --- | --- | --- | --- | --- | --- | --- | --- | --- | --- | --- | --- | --- |
|  | ***T*_M_ (^o^C)** | ***W* (^o^C)** | ***A***  **(µmol g^-1^)** |  | ***T*_M_ (^o^C)** | ***W* (^o^C)** | ***A***  **(µmol g^-1^)** |  | ***T*_M_ (^o^C)** | ***W* (^o^C)** | ***A***  **(µmol g^-1^)** |  | ***T*_M_ (^o^C)** | ***W* (^o^C)** | ***A***  **(µmol g^-1^)** |  | ***T*_M_ (^o^C)** | ***W* (^o^C)** | ***A***  **(µmol g^-1^)** |
| 0 (Blank) | - | - | - |  | 484 | 146 | 30 |  | 614 | 102 | 24 |  | 692 | 102 | 18 |  | - | - | - |
| 0.01 | 131 | 37 | 2 |  | 507 | 161 | 26 |  | 565 | 153 | 17 |  | 691 | 153 | 93 |  | 950 | 85 | 13 |
| 0.05 | 137 | 39 | 6 |  | 472 | 173 | 40 |  | 572 | 144 | 52 |  | 716 | 144 | 197 |  | 870 | 160 | 28 |
| 0.1 | - | - | - |  | 492 | 166 | 35 |  | 585 | 152 | 81 |  | 716 | 152 | 390 |  | 880 | 153 | 85 |
| 0.2 | - | - | - |  | 435 | 171 | 76 |  | 611 | 145 | 120 |  | 726 | 145 | 390 |  | 880 | 136 | 82 |
| 0.3 | - | - | - |  | 474 | 153 | 84 |  | 624 | 152 | 148 |  | 734 | 152 | 388 |  | 893 | 113 | 64 |

**
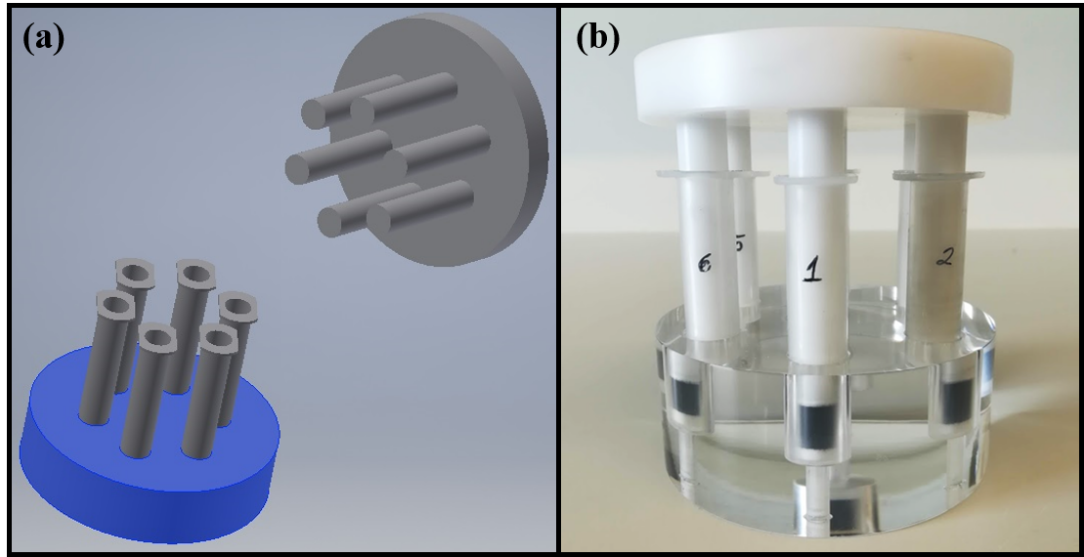
**

**Figure S1.** Schematic representation (a) and photograph (b) of the lab-scale packing device designed to prepare MWCNT cartridges (6 at a time).


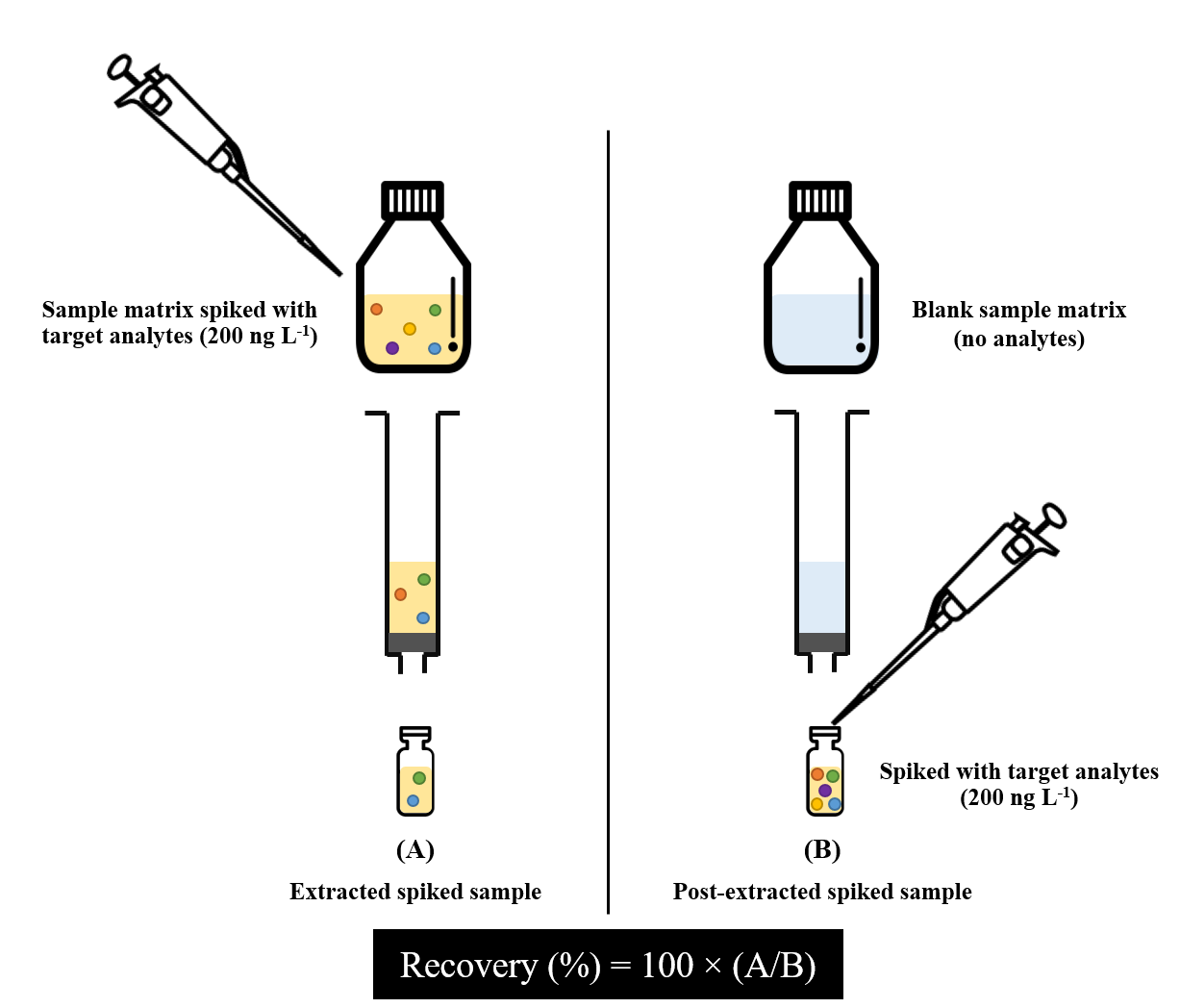
**Figure S2.** Schematic representation of the experimental procedure carried out to evaluate the extraction efficiency (i.e. recovery in %) of each SPE method.


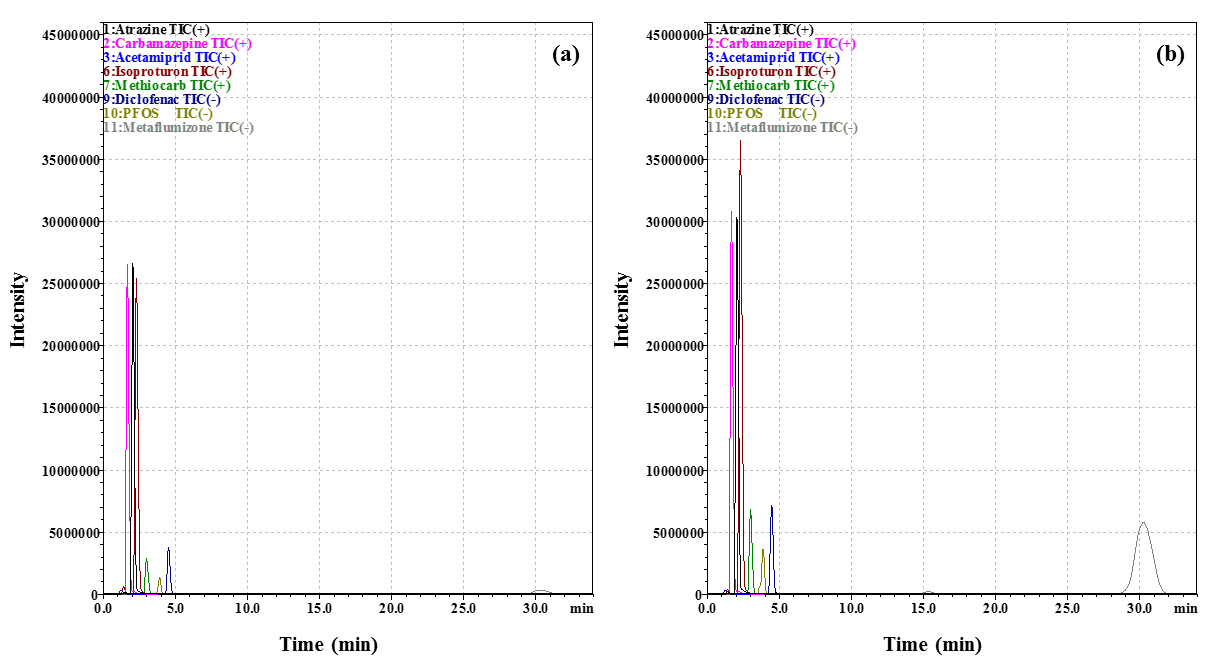


**Figure S3.**  Total Ion Current (TIC) chromatograms of the 8 target OMPs (200 ng L^-1^) in: (a) a SPE extract of a spiked sample; and (b) a post-spiked blank extract, using cartridges packed with MWCNTs (50 mg).

**Figure S4.** Deconvolution of **(a, c, e, g, i)** CO_2_ and **(b, d, f, h, j)** CO TPD spectra of MWCNTs subjected to hydrothermal treatment with different HNO_3_ concentrations. Dashed lines represent peaks assigned to strongly acidic carboxylic acids (SA), less acidic carboxylic acids (LA), carboxylic anhydrides (Can), lactones (Lac), phenols (Ph), carbonyls and quinones (CQ) and basic surface groups (Bas), such as pyrones and chromenes. Red lines represent cumulative peak fitting.


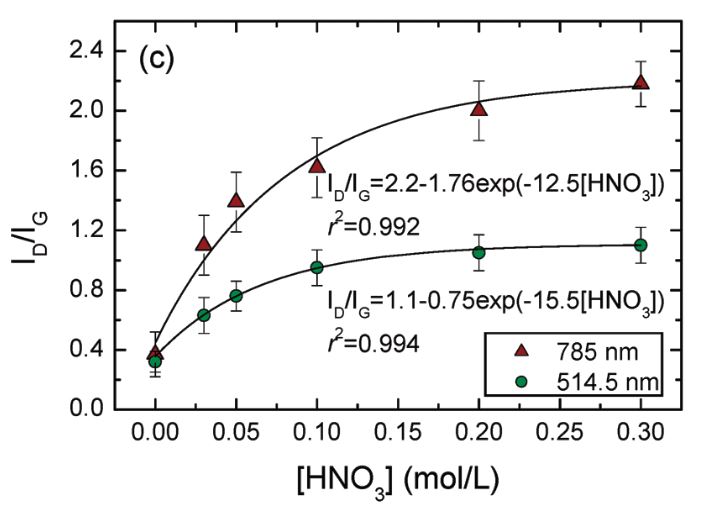


**Figure S5.** Intensity ratio of the D band relative to the G mode (*I*_D_/*I*_G_) obtained by Raman spectroscopy, as function of the total amount of evolved CO and CO_2_ determined by TPD when functionalizing single-walled carbon nanotubes with different HNO_3_ concentrations in the hydrothermal treatment. Lines designate the linear fit. Reprinted (adapted) with permission from The Journal of Physical Chemistry C, Vol. 115, G.E. Romanos, V. Likodimos, R.R.N. Marques, T.A. Steriotis, S.K. Papageorgiou, J.L. Faria, J.L. Figueiredo, A.M.T. Silva, P. Falaras, Controlling and Quantifying Oxygen Functionalities on Hydrothermally and Thermally Treated Single-Wall Carbon Nanotubes, 8534-8546. Copyright 2011, with permission of American Chemical Society.

**Figure S6.** N_2_ adsorption-desorption isotherms at -196 ^o^C of MWCNTs subjected to hydrothermal treatment with different HNO_3_ concentrations.


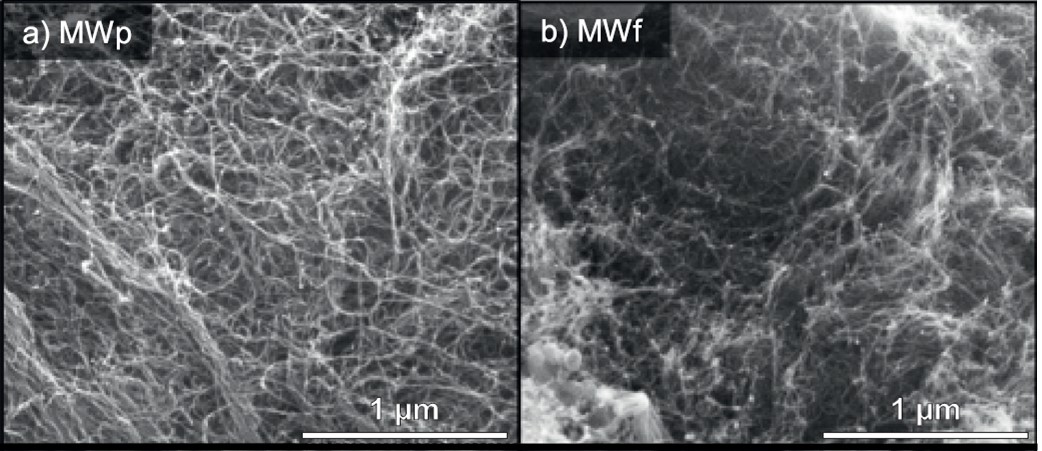


**Figure S7.** SEM micrographs of (a) pristine (MWp) and (b) functionalized (MWf) MWCNTs. Reprinted from The Journal of Membrane Science, Vol. 520, Sergio Morales-Torres, Carla M.P. Esteves, José L.Figueiredo, Adrián M.T. Silva, Thin-film composite forward osmosis membranes based on polysulfone supports blended with nanostructured carbon materials, 326-336, Copyright 2016, with permission from Elsevier [License number: 4945340204301].

**Figure S8.** Recoveries obtained for methiocarb as function of **(a)** [Ph]/*S*_BET_ and **(b)** [CQ]/*S*_BET_.

**References**

[1] M.O. Barbosa, A.R. Ribeiro, M.F.R. Pereira, A.M.T. Silva, Eco-friendly LC–MS/MS method for analysis of multi-class micropollutants in tap, fountain, and well water from northern Portugal, Analytical and Bioanalytical Chemistry, 408 (2016) 8355-8367.

[2] A.R. Ribeiro, M. Pedrosa, N.F.F. Moreira, M.F.R. Pereira, A.M.T. Silva, Environmental friendly method for urban wastewater monitoring of micropollutants defined in the Directive 2013/39/EU and Decision 2015/495/EU, Journal of Chromatography A, 1418 (2015) 140-149.

[3] Y.S. Al-Degs, M.A. Al-Ghouti, Preconcentration and determination of high leachable pesticides residues in water using solid-phase extraction coupled with high-performance liquid chromatography, International Journal of Environmental Analytical Chemistry, 88 (2008) 487-498.

[4] G. Min, S. Wang, H. Zhu, G. Fang, Y. Zhang, Multi-walled carbon nanotubes as solid-phase extraction adsorbents for determination of atrazine and its principal metabolites in water and soil samples by gas chromatography-mass spectrometry, Science of The Total Environment, 396 (2008) 79-85.

[5] Z.-g. Yu, Z. Qin, H.-r. Ji, X. Du, Y.-h. Chen, P. Pan, H. Wang, Y.-y. Liu, Application of SPE Using Multi-Walled Carbon Nanotubes as Adsorbent and Rapid Resolution LC-MS-MS for the Simultaneous Determination of 11 Triazine Herbicides Residues in River Water, Chromatographia, 72 (2010) 1073-1081.

[6] Q. Zhou, J. Xiao, W. Wang, G. Liu, Q. Shi, J. Wang, Determination of atrazine and simazine in environmental water samples using multiwalled carbon nanotubes as the adsorbents for preconcentration prior to high performance liquid chromatography with diode array detector, Talanta, 68 (2006) 1309-1315.

[7] A.H. El-Sheikh, J.A. Sweileh, Y.S. Al-Degs, A.A. Insisi, N. Al-Rabady, Critical evaluation and comparison of enrichment efficiency of multi-walled carbon nanotubes, C18 silica and activated carbon towards some pesticides from environmental waters, Talanta, 74 (2008) 1675-1680.

[8] L.M. Ravelo-Pérez, J. Hernández-Borges, M. Ángel Rodríguez-Delgado, Multiwalled carbon nanotubes as solid-phase extraction materials for the gas chromatographic determination of organophosphorus pesticides in waters, Journal of Separation Science, 31 (2008) 3612-3619.

[9] M.R. Hadjmohammadi, M. Peyrovi, P. Biparva, Comparison of C18 silica and multi-walled carbon nanotubes as the adsorbents for the solid-phase extraction of Chlorpyrifos and Phosalone in water samples using HPLC, Journal of Separation Science, 33 (2010) 1044-1051.

[10] Y.S. Al-Degs, M.A. Al-Ghouti, A.H. El-Sheikh, Simultaneous determination of pesticides at trace levels in water using multiwalled carbon nanotubes as solid-phase extractant and multivariate calibration, Journal of Hazardous Materials, 169 (2009) 128-135.

[11] L. Latrous El Atrache, M. Hachani, B.B. Kefi, Carbon nanotubes as solid-phase extraction sorbents for the extraction of carbamate insecticides from environmental waters, International Journal of Environmental Science and Technology, 13 (2016) 201-208.

[12] M. Dong, Y. Ma, E. Zhao, C. Qian, L. Han, S. Jiang, Using multiwalled carbon nanotubes as solid phase extraction adsorbents for determination of chloroacetanilide herbicides in water, Microchimica Acta, 165 (2009) 123-128.

[13] A. Speltini, M. Maiocchi, L. Cucca, D. Merli, A. Profumo, Solid-phase extraction of PFOA and PFOS from surface waters on functionalized multiwalled carbon nanotubes followed by UPLC–ESI-MS, Analytical and Bioanalytical Chemistry, 406 (2014) 3657-3665.

[14] M.A. Salam, R. Burk, Solid phase extraction of polyhalogenated pollutants from freshwater using chemically modified multi-walled carbon nanotubes and their determination by gas chromatography, Journal of Separation Science, 32 (2009) 1060-1068.

[15] Y.-q. Cai, Y.-e. Cai, S.-f. Mou, Y.-q. Lu, Multi-walled carbon nanotubes as a solid-phase extraction adsorbent for the determination of chlorophenols in environmental water samples, Journal of Chromatography A, 1081 (2005) 245-247.

[16] J. Ma, R. Xiao, J. Li, J. Yu, Y. Zhang, L. Chen, Determination of 16 polycyclic aromatic hydrocarbons in environmental water samples by solid-phase extraction using multi-walled carbon nanotubes as adsorbent coupled with gas chromatography–mass spectrometry, Journal of Chromatography A, 1217 (2010) 5462-5469.

[17] W.-D. Wang, Y.-M. Huang, W.-Q. Shu, J. Cao, Multiwalled carbon nanotubes as adsorbents of solid-phase extraction for determination of polycyclic aromatic hydrocarbons in environmental waters coupled with high-performance liquid chromatography, Journal of Chromatography A, 1173 (2007) 27-36.

[18] B. Lalović, T. Đurkić, M. Vukčević, I. Janković-Častvan, A. Kalijadis, Z. Laušević, M. Laušević, Solid-phase extraction of multi-class pharmaceuticals from environmental water samples onto modified multi-walled carbon nanotubes followed by LC-MS/MS, Environmental Science and Pollution Research, 24 (2017) 20784-20793.

[19] S. Dahane, M.D. Gil García, M.J. Martínez Bueno, A. Uclés Moreno, M. Martínez Galera, A. Derdour, Determination of drugs in river and wastewaters using solid-phase extraction by packed multi-walled carbon nanotubes and liquid chromatography–quadrupole-linear ion trap-mass spectrometry, Journal of Chromatography A, 1297 (2013) 17-28.
